# Personalized health predictions challenge existing insurance frameworks: Time to revisit life and disability insurance regulation?

**DOI:** 10.1002/jgc4.70206

**Published:** 2026-04-16

**Authors:** Max Rensink, Maartje Schermer, Ineke Bolt

**Affiliations:** ^1^ Department of Public Health – Medical Ethics, Philosophy, and History of Medicine Group Erasmus Medical Centre Rotterdam The Netherlands; ^2^ Erasmus School of Health Policy and Management Erasmus University Rotterdam The Netherlands

**Keywords:** ethics, Huntington's disease, insurance underwriting, life insurance, neurogenerative diseases, personalized predictions, polygenic risk scores, presymptomatic genetic testing

## Abstract

Personalized health predictions are developing rapidly across a wide range of diseases, challenging the existing legal frameworks for life and disability insurance. These frameworks were originally developed with a focus on presymptomatic genetic testing for severe monogenic diseases such as Huntington's disease. The rapid expansion of personalized medicine raises questions about whether existing legal frameworks remain adequate. This paper examines the underlying ethical values of current legal frameworks that rely on financial thresholds to balance stakeholders' interests and explores how the growing availability of personalized predictions may give rise to ethical tensions. We argue that it is important to find a renewed balance between stakeholders' interests, to evaluate whether the concept of genetic exceptionalism is still meaningful in light of personalized predictions, and to consider how legislation can be reformulated to encompass personalized predictions and ensure solidarity. Furthermore, the potential for inaccuracies and misinterpretation of personalized predictions should be addressed. Our analysis highlights several ethical tensions that current legal frameworks may not be well equipped to address as personalized prediction technologies evolve. We therefore conclude that re‐evaluating the legal frameworks underpinning life and disability insurance underwriting is both timely and necessary.

**What is known about this topic**

Personalized health predictions are developing rapidly across a wide range of diseases, and concerns have been raised about their potential impact on insurance underwriting. Current national legislation on insurance underwriting reflects a balance between the interests of individuals at risk for monogenic diseases and those of life insurers and, indirectly, other policyholders.

**What this paper adds to the topic**

This paper first argues that personalized predictions challenge the concept of genetic exceptionalism, as personalized predictions often combine genetic and non‐genetic data. Second, individual health risks become more transparent and quantifiable, which may threaten a broad conception of solidarity that underpins many existing legal frameworks. Third, personalized prediction results are typically less accurate and more complex than traditional genetic test results. For these reasons, this paper calls for a re‐evaluation of the legal frameworks underpinning life and disability insurance underwriting in which the interests of individuals with elevated disease risks are adequately recognized.

## 1 INTRODUCTION

*Personalized medicine* is developing rapidly across a broad spectrum of diseases, from cardiovascular diseases to cancer and diabetes. It aims to tailor risk assessments and treatment strategies to the individual, using various kinds of biomarkers, genetic markers, and other personal data (Lan et al., 2024; Louis & Roche, 2017; Stefanicka‐Wojtas & Kurpas, 2023). These developments are promising, as they may lead to more individualized information and effective treatments, but at the same time, they introduce new layers of complexity.

One area where personalized medicine causes significant changes and increases complexity is the field of genetic testing (Abul‐Husn et al., 2014; Claussnitzer et al., 2020). Traditionally, genetic testing was mainly performed for monogenic (or *Mendelian*) diseases, but new technologies such as whole genome sequencing and polygenic risk scores (PGS) make it increasingly possible to gain insight into individual disease risk across multiple diseases. PGS, for instance, estimate an individual's risk for complex conditions by analyzing “multiple disease‐associated common genetic variants” (Yanes et al., 2024). PGS has broad clinical applications, potentially offering risk predictions for a wide range of conditions (Yanes et al., 2020, 2024). Another example of how personalized medicine transforms genetic testing is the development of biomarker‐based prediction models for monogenic neurodegenerative conditions. While testing for monogenic diseases has traditionally only provided information about carriership of a specific mutation, a Dutch research consortium is currently working on developing individual onset and progression predictions for Huntington's disease (HD) and Spinocerebellar Ataxias (CureQ, 2022), which are burdensome neurodegenerative diseases for which no disease‐modifying treatments are available. Onset and progression predictions aim to provide information that is specifically tailored to the individual and to inform individuals about their future disease trajectory.

By offering more individualized information, personalized medicine not only changes genetic testing but may also challenge existing life and disability insurance legislation. Many of the legal frameworks regarding life and disability insurance have been shaped with particular attention to monogenic diseases, specifically HD (Popma & Westerveld, 2007). These legislations, which are private forms of insurance, regulate the conditions under which information on carrier status must be shared with insurers (Godard et al., 2003).

One prominent regulatory strategy is to set financial thresholds that determine when genetic information may be requested or used. Setting financial thresholds represents one of several international policy approaches for dealing with genetic discrimination, alongside moratoria, explicit rights‐based prohibitions, and approaches that delegate decisions to an independent scientific board or leave them to insurers themselves (Joly et al., 2010). The goal of setting financial thresholds is to balance the interests of individuals undergoing presymptomatic genetic testing, who wish to learn whether they will develop disease symptoms in the future, and those of insurers, who wish to prevent adverse selection to maintain profitability and keep premiums acceptable for all policyholders (Nederlandse overheid [Dutch government], 1998; Yanes et al., 2024). The rise of personalized medicine may disrupt the balance between stakeholders in current legal frameworks, because personalized predictions offer more individualized health information than traditional genetic tests.

For instance, Yanes et al. (2024) state that “PGS has the capacity to change the way insurers consider and use genetic information” (page 3) and are concerned that insurers will use PGS to deny coverage. In contrast, while acknowledging the risk for genetic discrimination, Linnér and Koellinger (2022) argue that PGS could negatively impact insurers because PGS carries the potential for information asymmetry and adverse selection. Biomarker‐based onset prediction models for monogenic diseases may disrupt the balance between stakeholders in a similar way. Therefore, it is important to assess the current balance between stakeholders and analyze the ethical values underpinning legal frameworks to determine which values may be threatened due to the rise of personalized medicine in general, and due to personalized predictions in particular.

In this article, we focus on financial thresholds as a regulatory mechanism for balancing the interests of individuals and insurers. While various international regulatory models use alternative approaches (Joly et al., 2010), the Dutch approach—among others, most of which are European—relies on financial thresholds, and the balancing of stakeholder interests is addressed directly in legislation itself, rather than, for example, left to a scientific board or insurers. The explicit explanation in legislation of why the balance between stakeholders requires financial thresholds sheds light on the underlying normative choices. Analyzing the Dutch approach therefore allows us to provide insights in the ethical values embedded in legal frameworks and to discuss the ethical tensions that may arise with the growing availability of personalized predictions. First, we discuss the legal balance between competing interests of stakeholders and provide an example of how this balance may be affected by personalized predictions. After that, we discuss the validity of the idea of genetic exceptionalism. Subsequently, we discuss two notions of solidarity and argue that, in the era of personalized medicine, it is necessary to explore how solidarity can be adequately anchored in legislation. Lastly, we discuss the potential consequences of the lower accuracy and higher complexity of personalized predictions compared to traditional genetic test results. We illustrate these challenges using examples drawn from PGS and onset prediction for HD. Our ethical analysis highlights several ethical tensions that current legal frameworks may not be well equipped to address when personalized prediction technologies evolve. We therefore conclude that a re‐evaluation of the legal frameworks underpinning life and disability insurance underwriting is both timely and necessary.

## 2 COMPETING INTERESTS BETWEEN STAKEHOLDERS

Current legislation seeks to balance the interests of individuals who undergo presymptomatic genetic testing and insurers who aim to provide proportionate premiums to all the insured. For asymptomatic individuals who undergo genetic testing for a monogenic condition, privacy protection is very important. This is not only because health information is sensitive information but also because individuals may otherwise refrain from testing due to concerns about negative (financial) consequences for themselves or their family members (Erwin et al., 2010; Popma & Westerveld, 2007). In addition, it could create undue pressure to undergo testing, as a negative test result might lead to lower insurance premiums. This would undermine the “right not to know”—“the right not to be informed against one's expressed wishes” (Popma & Westerveld, 2007). Insurers rely on access to personal (health) data to assess risk accurately (Ossa & Towse, 2004). Genetic information enhances their ability to predict premature death or disability, allowing for more precise premium differentiation. From the insurer's perspective, information asymmetry, where the applicant knows their genetic status but the insurer does not, is problematic and potentially leads to adverse selection (Ossa & Towse, 2004). Adverse selection occurs when individuals at high risk seek insurance while individuals at low risk opt out, which potentially increases overall costs and raises standard premiums for everyone (Cutler & Zeckhauser, 1998).

Personalized predictions require the collection of more health data, reveal increasingly detailed personal health information, and may therefore increase the need for privacy protection. However, insurers may be concerned that personalized predictions increase the risk for information asymmetry and thus adverse selection. For example, although not yet possible, a personalized onset prediction for HD might indicate that a mutation carrier is likely to develop disease symptoms between ages 50 and 55, which the individual can take into account when applying for life or disability insurance. Insurers may want access to this information to prevent adverse selection. Whether adverse selection is a real risk when personalized predictions become more widely available is uncertain, but these competing interests may intensify and potentially disrupt the existing balance in legislation. It is therefore important to explore whether and how legislation should be adjusted to ensure a fair balance.

## 3 VALIDITY OF GENETIC EXCEPTIONALISM

The concept of genetic exceptionalism lies at the roots of current (European) legal frameworks, as many legal frameworks draw a sharp line between genetic and non‐genetic information (Soini, 2012). This distinction is known as *genetic exceptionalism*, which holds that genetic data are inherently more personal and fundamentally different from other health information, thus warranting special protection (Murray, 2019). However, the concept of *genetic exceptionalism* has faced wide criticism, as it has been argued that genetic information is neither unique nor morally distinct from other health information, and that portraying it as such is not productive and oversimplifies the issues (see, for example, Murray (2019), Garrison et al. (2019), and Kakuk (2008)).

Personalized predictions increasingly combine genetic and non‐genetic factors, and it is therefore questionable whether the traditional distinction between genetic and non‐genetic information is still meaningful. Onset prediction in HD may for instance integrate multiple data sources, including information about the genetic mutation, magnetic resonance imaging (MRI) findings, blood biomarkers such as neurofilament light chain, and clinical assessments (CureQ, 2022). These hybrid onset prediction models may thus create regulatory ambiguity: does such blended information still qualify as “genetic”? Similar uncertainty exists regarding PGS, and Yanes et al. (2024) have already argued for extending the existing regulations to cover PGS as well. It seems therefore necessary to evaluate whether and how current legal regulations can be reformulated to encompass these types of personalized predictions.

## 4 SOLIDARITY UNDER PRESSURE

When legal frameworks for genetic testing were established, discussions took place on different notions of solidarity to prevent genetic discrimination (Van Hoyweghen, 2010). One notion of “solidarity” is *chance solidarity*, more commonly known as actuarial fairness (Lehtonen & Liukko, 2011). This implies that “each insured individual participates in the risk pool with a share that equals his or her *personal likelihood* of causing damage to the *whole risk pool*” (Lehtonen & Liukko, 2011) (page 38, original emphasis). In this technical conception of solidarity, insurers group individuals in risk classes based on various personal and health‐related data in order to inform underwriting decisions. A more social notion of solidarity is discussed by Prainsack and Buyx (2012), who conceptualize solidarity as voluntary, relational practices based on similarities. They define it as “shared practices reflecting a collective commitment to carry ‘costs’ (financial, social, emotional, or otherwise) to assist others” (Prainsack & Buyx, 2012) (page 346). In this view, solidarity goes beyond actuarial risk sharing: it is a moral commitment to support others who are less fortunate. In many European countries, health and life insurances are considered a basic social good (Van Hoyweghen, 2010). Although health insurance may appear more critical, life insurance often functions as a prerequisite for accessing other social goods, such as mortgages. The legal ban on life insurers requesting genetic test results below a defined financial threshold, implemented in several countries, reflects the broader notion of solidarity by ensuring that the financial costs for individuals with a high risk of future disease are shared collectively.

Personalized predictions increasingly make personal risk identifiable, which may encourage a more technical conception of solidarity. The ability to classify individuals into more detailed risk classes may threaten the broader conception of solidarity that currently underpins existing legislations. It is therefore important to safeguard a broader interpretation of solidarity in legislation, in order to support those who are less fortunate and to protect them against unfair “genetic” discrimination. In the era of personalized medicine, acknowledging that “we all carry data” may furthermore stimulate new forms of solidarity (Prainsack & Van Hoyweghen, 2020).

## 5 LOWER ACCURACY AND HIGHER COMPLEXITY

Presymptomatic genetic testing for certain monogenic conditions is usually highly accurate and relatively straightforward to perform (e.g., Kremer et al. (1994)). Despite the high accuracy of presymptomatic tests, the consequences of testing for eligibility for life and disability insurance are often perceived as complex, leading to misunderstandings that contribute to hesitancy toward undergoing presymptomatic testing. In the Netherlands, for example, many individuals at risk fear that a test result indicating increased genetic risk could limit access to a mortgage, even though this may not be the case (Bunnik, 2012; de Kwant, 2015). These misunderstandings and fears can drive misinformed decisions and may unnecessarily lead individuals to refrain from testing.

Both the accuracy and complexity of personalized predictions require attention. First, personalized predictions likely have a lower accuracy than current presymptomatic genetic testing, because they must account for additional (genetic) variables and disease modifiers (Gusella & MacDonald, 2009; Lee et al., 2015), whereas presymptomatic testing targets a single pathogenic gene. In addition, most genetic datasets are derived from individuals of European ancestry (Fatumo et al., 2022; Martin et al., 2019), which may limit predictive accuracy for other populations and may further exacerbate existing health disparities. Concerns have been raised about the accuracy and representativeness of PGS (Yanes et al., 2024), and these concerns extend to other forms of personalized predictions (Najafzadeh et al., 2013). Under conditions of uncertainty due to low(er) accuracy, insurers may adopt conservative assumptions to minimize financial risk, such as basing coverage decisions on the earliest predicted onset. This can result in higher premiums or even exclusion from coverage for individuals at increased risk of disease.

Second, personalized predictions introduce higher complexity compared to presymptomatic genetic testing. Rather than a binary test result, personalized predictions are likely to provide more complex outcomes, such as estimated age ranges for disease onset or probabilities of developing a specific condition. Consider a (hypothetical) personalized predicted disease onset for an HD carrier between ages 48 and 53 with 90% accuracy. Such information is more difficult to understand for individuals and harder for healthcare professionals to communicate than a binary test result. Personalized predictions may therefore contribute to misunderstandings.

In conclusion, the lower accuracy and higher complexity of personalized predictions compared to traditional presymptomatic genetic testing may result in conservative coverage decisions and exacerbate existing misunderstandings. It is therefore important to ensure adequate accuracy and clear communication regarding the potential consequences of receiving personalized predictions for insurance underwriting.

## 6 CONCLUSION

Current life and disability legislation that sets financial thresholds for requesting genetic information reflects a balance between the interests of individuals at risk for monogenic diseases and those of insurers and, indirectly, other policyholders. This balance is traditionally grounded in the concept of genetic exceptionalism and underpinned by notions of solidarity. Personalized predictions, however, challenge the concept of genetic exceptionalism. Since personalized predictions often integrate both genetic and non‐genetic data, discussion on whether genetic exceptionalism remains suitable within existing regulatory frameworks is required. Furthermore, existing legislations are often based on a broad interpretation of solidarity. Yet, the growing transparency and quantifiability of individual risk may cause a shift away from this broader conception of solidarity. Further empirical, social, and ethical‐philosophical research is needed to explore how solidarity can be meaningfully integrated into regulatory frameworks. Finally, test results of personalized predictions tend to become less accurate and more complex than traditional presymptomatic genetic testing. It is therefore important to reduce inaccuracies and address the risk of misinterpretation of personalized predictions. As personalized prediction technologies evolve rapidly, awareness of these potential concerns among genetic counselors is warranted. The developments of personalized predictions call for a re‐evaluation of existing life and disability insurance legislation.

## AUTHOR CONTRIBUTIONS

All authors contributed to the conceptualization of the paper. MR drafted the first version. IB and MS commented on and reviewed the manuscript. MR revised the manuscript. All authors approved the final version of the manuscript.

## CONFLICT OF INTEREST STATEMENT

All authors declare no competing interests.

## ETHICS STATEMENT

Human studies and informed consent: Informed consent is not applicable, as this study does not involve participants. Ethical approval was not required. No non‐human animal studies were carried out in this study.

## Data Availability

Data sharing is not applicable to this article as no datasets were generated or analyzed during the current study.
